# Wavelength-Dependent Electrical Readout of Spin Ensembles in a Thin-Film SiC-on-Insulator Platform

**DOI:** 10.1021/acs.nanolett.5c05971

**Published:** 2026-04-20

**Authors:** Alexander Zappacosta, Ben Haylock, Paul Fisher, Naoya Morioka, Robert Cernansky

**Affiliations:** † Institute for Quantum Optics, 9189 Ulm University, Ulm 89081, Germany; ‡ Institute for Chemical Research, 12918 Kyoto University, Uji, Kyoto 611-0011, Japan; ¶ Center for Spintronics Research Network, Institute for Chemical Research, Kyoto University, Uji, Kyoto 611-0011, Japan

**Keywords:** silicon carbide, quantum electronics, photoelectric detection, silicon vacancy, wavelength dependence, thin-film silicon carbide-on-insulator

## Abstract

We report electrical spin-state readout and coherent control of an ensemble (∼540) of silicon vacancies (V_Si_^–^) in a silicon carbide-on-insulator (SiCOI) platform, with excitation wavelengths from 780 to 990 nm, demonstrating for the first time spin-state readout well beyond the zero phonon line of the V2 V_Si_^–^. By implementing photoelectrical detection of magnetic resonance in thin-film SiCOI, we merge a scalable spin readout technique requiring no collection optics, together with a promising platform for future scalable and CMOS-compatible integrated photonics. Furthermore, we provide a comparison of optical and electrical readout between bulk silicon carbide (SiC) and thin-film SiCOI, revealing that our thin-film processing has a measured *T*_2_ coherence time of ≈7 μs, similar to that in the bulk SiC. These results extend the capabilities of SiCOI toward electronic and spin-based devices for scalable quantum technologies over a wide range of excitation wavelengths.

## Graphical abstract

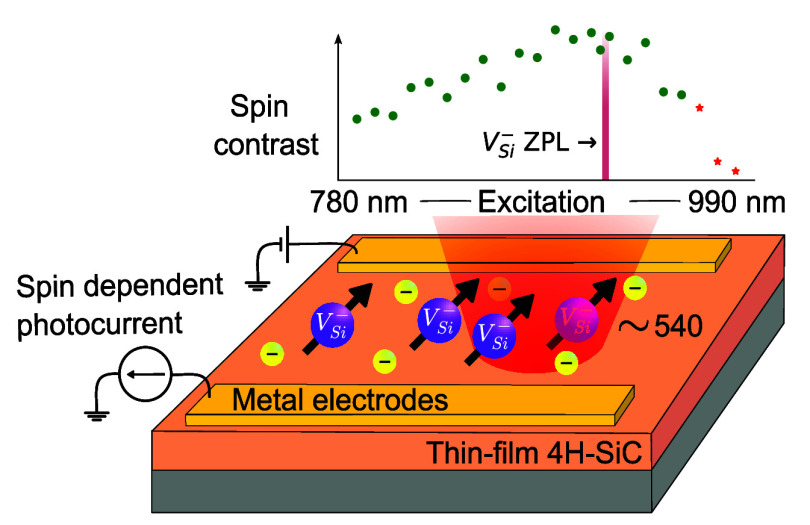

Silicon carbide (SiC) is a CMOS-compatible semiconducting material that hosts a variety of solid-state spin systems including divacancy,^1–3^ silicon vacancy (V_Si_^–^),^4–6^ and nitrogen vacancy.^7–9^ These systems allow for optical initialization, control, and readout with coherence times exceeding 1 ms,^2^ making them excellent candidates for a variety of quantum technologies^10,11^ including sensing^12–17^ and communication.^18,19^ Current bulk SiC experiments utilize optically detected magnetic resonance (ODMR), limiting their scalability due to large amounts of photon collection optics including wavelength filtering and photodiodes.

Photoelectrical detection of magnetic resonance (PDMR)^20^ enables full integration of detection directly onto the SiC surface, extracting charges from ionized spin systems for electrical spin-state readout. This is possible with patterned electrodes because their physical size can bypass the optical diffraction limit using standard lithography techniques, making PDMR an excellent solution for scaling up spin-based detection systems. Furthermore, photoelectrical charge-carrier generation does not saturate at high excitation powers,^20^ enabling higher laser intensities and thus improved readout fidelity compared to optical measurements.^21,22^

Electrical spin readout has been demonstrated for a wide range of materials such as diamond for singles^23^ and ensembles^20^ of nitrogen vacancies, hexagonal boron nitride for negatively charged boron vacancies,^24^ gallium arsenide quantum dots,^25^ and phosphorus donor electrons in silicon.^26^ However, PDMR in SiC has only been demonstrated for bulk experiments with a single^21^ and an ensemble^27,28^ of silicon vacancies including a SiC n^+^–p junction diode^29^ and metal–oxide–semiconductor field-effect transistor (MOSFET).^30^ Implementing PDMR in a platform that supports integrated photonics is a step toward scalable spin initialization and detection systems, reducing the need for bulk optics and photodetectors.

SiC integrated photonics require a thin SiC layer on the order of several microns, bonded to an insulating substrate, such as silicon dioxide (SiO_2_). This combination is referred to as silicon carbide on insulator (SiCOI). Photonics in SiCOI have already demonstrated low-loss optical devices,^31,32^ ultrahigh-*Q* SiC photonic crystal nanocavities,^33^ fast optical modulators,^34^ and integration with defects,^18^ making SiCOI a promising platform for scalable quantum technologies.^35^ Implementing PDMR on a thin-film SiCOI platform thus provides an elegant solution to scale up hundreds of on-chip spin-state detection systems without the need for collection optics. Combining PDMR and SiCOI would be particularly useful in applications where only local readout and no photon entanglement is required, such as waveguide-based quantum sensing^12^ and spin–spin-entangled computation.^36^ Furthermore, waveguides enhance light–matter interactions by efficiently increasing the interaction volume, thereby enabling efficient readout of PDMR, reducing the required laser power, and making it more compatible for biosensing applications.

In this work, we demonstrate for the first time room temperature PDMR of a small ensemble (∼540) of V2 silicon vacancies in a 4H-SiCOI platform. Furthermore, we compare optical and electrical readouts in bulk and thin-film SiC, establishing that our fabrication process has no significant effect on the PDMR signal and *T*_2_ coherence time. Finally, we report electrical spin-state readout across wavelengths ranging from 780 to 990 nm. Demonstrating for the first time electrical spin-state readout well beyond the zero phonon optical transition energy (ZPL) of V2 V_Si_^–^ and having optimal contrast within a wide range of wavelengths around the ZPL ± 20 nm.

The silicon vacancy at the cubic lattice site V_Si_^–^ consists of five active electrons: four from dangling bonds surrounding the vacancy and one captured electron. This leads to an optically active ground state with spin quartet configuration (*S* = ^3^/_2_).^6^ The zero phonon optical transition energy (ZPL) of V2 V_Si_^–^ is 1.35 eV (917 nm).^37^ Off-resonant laser excitation promotes the ±|^3^/_2_⟩ and ±|^1^/_2_⟩ spin states to the excited state (Figure 1). Relaxation occurs either through nonradiative decay via an intersystem crossing (ISC) into a metastable state (MS) or via spin-conserving radiative decay, yielding room-temperature broadband phonon-assisted emission from 850 to 1000 nm.^38^ Off-resonant laser excitation pumps the ±|^1^/_2_⟩ state, which preferentially decays through ISC (dark), while ±|^3^/_2_⟩ decays radiatively (bright). These spin sublevels are separated by a zero-field splitting of 2*D*_gs_0__ ≈ 70 MHz. Through simultaneous laser illumination and resonant radio-frequency (RF) driving at 2*D*_gs_0__, an optical spin contrast is observed, defined as a percentage (%) given by

1 $$\left(\frac{S_{\mathrm{RF}}}{S_{0}}-1\right)\times 100$$

where *S*_RF_ and *S*_0_ are the detected signal magnitudes with and without RF drive, respectively. In the case for optical detection, *S* is the measured count rate.

**Figure 1:**
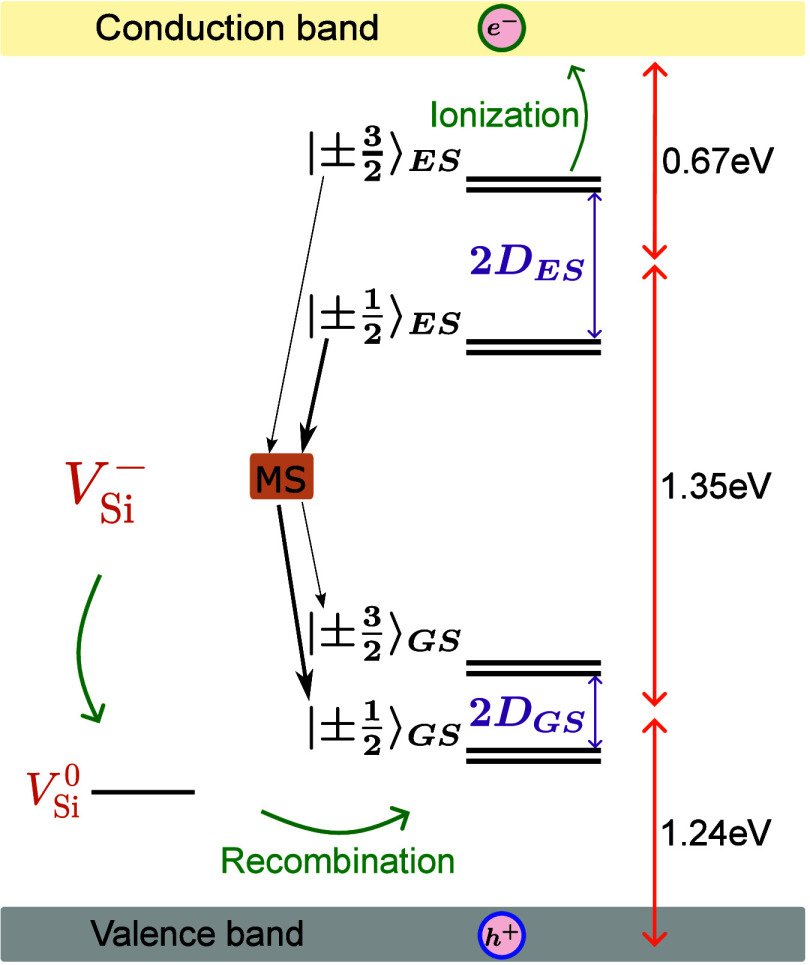
Spin quartet energy states of V_Si_^–^ with an optical transition of 1.35 eV between the ground and excited states of V_Si_^–^. Green arrows indicate the charge cycling. A photon-induced photocurrent from the defect is enabled by electron generation in the conduction band via two-photon absorption and photon-induced hole generation in the valence band.

An external magnetic field *B*_0_ along the SiC crystal *c* axis lifts the spin-state degeneracy, providing a field-dependent Zeeman splitting given by 2*D*_gs_ = 2*D*_gs_0__ ± γ*B*_0_, where γ is the electron gyromagnetic ratio. By tuning the RF frequency to 2*D*_gs_, one can address the individual spin states of the defect.

Electrical readout of the spin state utilizes the technique of PDMR. This is possible by first exciting the electrons within V_Si_^–^ to the excited state (1.35 eV), and a second photon ionizes V_Si_^–^ to the neutral charge state V_Si_^0^, promoting an electron to the conduction band (CB).^21^ Recently, it has been proposed that an ionization path through the doubly negative charge state V_Si_^2–^ might be a possible mechanism for electrical detection.^39^ Finally, absorption of a third photon generates a hole in the valence band (VB). This charge cycling process through the excited state^21^ forms the basis for a spin-dependent photocurrent (Figure 1).

An electrical spin-state contrast is possible by driving an RF frequency matching 2*D*_gs_, increasing the ±|^3^/_2_⟩ population. This inherently decreases the metastable state occupation, and therefore more electrons are available for ionization to the conduction band, leading to a measurable increase in the photocurrent. Electrically detected spin-state contrast is calculated using eq 1, the same as optical measurements; however, signal *S* is the magnitude of the measured photocurrent.

Electrical readout also collects charge contributions from other defects and free carriers. Therefore, the total detected photocurrent under laser illumination is the sum of all charge contributions, including from V_Si_^–^, and is referred to as the laser-induced background.

Commercially available research-grade high-purity-semi-insulating (HPSI) 4H-SiC was bonded to a SiO_2_ insulator with silicon as a substrate. The bonded SiC was ground, polished, and etched down to a thickness of 1.3 μm; the three-layer device is shown in Figure 2D. Gold electrodes of 150 nm with a 10 nm chromium adhesion layer were patterned on the SiC surface using standard optical laser lithography. The electrodes were covered with optically transparent hydrogen silsesquioxane (HSQ) to mitigate environmental fouling by moisture and airborne particulates, leaving patterned windows only for bonding pads and the 50 μm enamelled copper RF line shown in Figure 2C. The gold electrodes were wire bonded with aluminum wire to a 50 Ω impedance-matched printed circuit board and biased with a linear power supply for charge extraction. The photocurrent was measured using a 1 kHz 10^9^ V/A transimpedance amplifier (TIA) directly connected to a 2 Ms/s analog data acquisition device (DAQ). The experimental ground was an isolated unanodized aluminum breadboard directly connected to the building ground in which all electronic devices are shared. This configuration measured a dark current of 80 pA and a measurement noise of 20 fA with a bias of 8 V. Zeeman energy-level splitting was induced with a permanent magnet placed underneath the sample, and all measurements were performed at room temperature.

**Figure 2:**
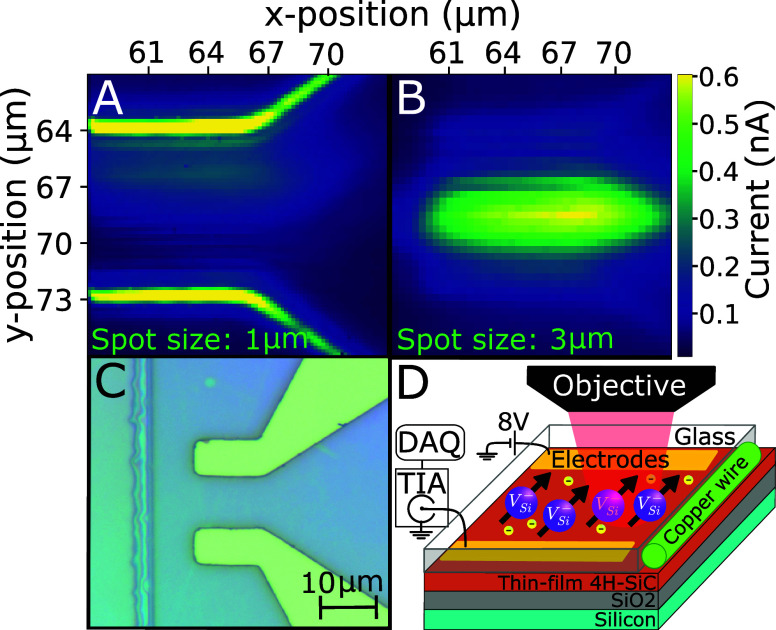
Confocal photocurrent scans (A and B) of the 4H-SiCOI device (D) at 23 mW, 10 V bias, and 870 nm. The 1 μm spot size (A) has a maximum photocurrent of 1.2 nA at the edge of the electrodes, and the 3 μm spot size (B) has a maximum photocurrent of 0.8 nA between the electrodes. The larger spot size demonstrates a broader distribution of photocurrent extraction. A microscope photograph (C) of the electrode device covered in HSQ.

Optical excitation was performed on the surface between the electrodes using a 662–1050 nm wavelength selective Ti:Sa continuous-wave ring laser. When the focal position of the objective is lowered, the laser spot size on the SiC surface is increased, resulting in different current extraction regimes. An electrical confocal image was constructed by scanning across a 10 μm × 10 μm region in 300 nm steps using 23 mW of a 890 nm pump laser light with 10 V biased electrodes and measuring the photocurrent at each point for 200 ms, as shown in parts A and B of Figure 2 for spot diameters of 1 and 3 μm, respectively. Figure 2A shows a maximum photocurrent of 1.2 nA along the electrode edges. Figure 2B is defocused into the sample, demonstrating a larger, more uniform distribution of charge collection with a maximum photocurrent of 0.8 nA between the electrodes and less variance in the photocurrent (Figure S4).

The number of measured silicon vacancies in each focus regime was estimated by considering the photon emission rate of 3–4 kc/s for a single V2 V_Si_^–^ measured in an identical optical collection setup used in ref 12 with a 805 nm long-pass dichroic. A 1-μm-diameter laser spot focused on the SiCOI surface collected a maximum measured photon emission of 80 kc/s without HSQ cover and with a 900 nm long pass, cutting approximately half the phonon emission sideband,^38^ equating to ∼40–50 spins. A spot size (Airy diameter) of 3 μm equates to 9 times larger illumination area; therefore, in the defocused regime, we estimate an electrical signal comprised of approximately 540 V_Si_^–^ including the possible contribution of higher-order Airy rings (∼20%) generating a photocurrent. The estimation does not account for the possibility that more defects may be illuminated and may contribute to the photocurrent than are illuminated and collected in the optical signal.

Pulsed spin control measurements were performed by sending a continuous RF signal through a fast RF switch and amplifying the switched RF signal for the spin control. The AOM, RF switch, and DAQ readout were triggered with a GHz TTL pulse sequence streamer. Pulsed PDMR and Rabi sequences were continuously streamed for each frequency or RF duration, with an 8 Hz square envelope modulating the RF amplitude for signal and reference measurements. The laser pulse duration was set to 1338 ns, with the MW pulse leading edge set at 1000 ns plus the maximum MW pulse duration, after each laser pulse leading edge, varying the duration of the MW pulse by extending the trailing edge and keeping the time between laser pulses constant, ensuring a consistent integer number of laser pulses for each envelope period. The analog voltage from the TIA output was collected every 500 ns by DAQ, integrating a window within the 8 Hz envelope half-period for the highest signal-to-noise ratio (SNR). For optical measurements, laser and emission were filtered with a 900 nm long-pass dichroic and further filtered with a long-pass filter before collection with a fiber-coupled avalanche photodiode (APD). Photon counts were collected for a 300 ns window at the beginning of each laser pulse, ensuring that the signal is proportional to the state population, with a delay accounting for AOM rise time.

Pulsed PDMR was successfully demonstrated in the 4H-SiCOI platform (Figure 3D) and in a 500 μm bulk sample (Figure 3A) from the same original 4H-SiC wafer, with identical electrodes at 890 nm, 45 mW, and 8 V bias and covered with the same transparent layer of HSQ. The bulk electrical PDMR has a contrast of 0.008% and the thin-film contrast is 0.025%, calculated using eq 1. PDMR results are fitted to two Lorentzian functions, three for the case of the negative dip in the SiCOI measurement, which has been observed in previous studies and possibly due to excitation of the V1′ silicon vacancies with minimal change to the relative V2 signal.^12,40^

**Figure 3:**
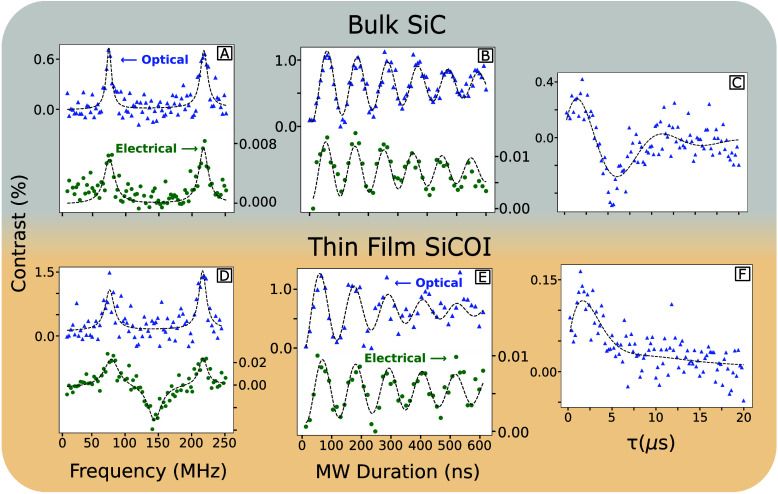
Measured pulsed PDMR and pulsed ODMR in a 1.3 μm thin-film SiCOI device (D) and bulk 500 μm sample (A). All electrical measurements performed at 8 V bias, 890 nm, and 45 mW. Electrical Rabi oscillations are observed with contrast ≈ 0.01% for both bulk (B) and thin film (E). Optical *T*_2_ Hahn echo measurement fitted with an ESEEM function (C and F) demonstrating coherence times of ≈7 ± 0.5 μs for the thin film and bulk.

Electrical Rabi oscillations were also observed for both bulk (Figure 3B) and thin film (Figure 3E) under the same measurement conditions, with a contrast of 0.01%, confirming PDMR readout of the silicon vacancies within the SiCOI layer after grinding, polishing, and inductively coupled plasma (ICP) etching down to 1.3 μm (suitable for waveguides). The corresponding Rabi oscillations were fitted to exponentially damped sinusoidal functions using least-squares regression. Laser powers from 30 to 50 mW showed no measurable change in electrical Rabi contrast. The measured contrast was limited by the density of spin ensemble and background photocurrent from non-V_Si_^–^. The highest reported PDMR contrast can be up to 0.4% for single silicon vacancies^21^ with reduction of background photocurrent from partially insulating electrodes. Furthermore, SiCOI demonstrated a small decrease in the background photocurrent compared to the bulk (Figure S1). Contrast is further dependent on the laser position inside the electrodes and required positional stabilization for consistent results (Figure S3).

For optical measurements, the laser was under fully focused conditions, maximizing photon counts for both the thin film and bulk, locked to an ensemble inside the electrodes within the same approximate position as electrical measurements. A laser power of 2 ± 0.5 mW was used, which was close to the saturation point of the defects. The measured optical Rabi contrast is around 1% for the thin film and bulk.

Electrical readout exhibited electrical spin contrast 75 times lower than optical spin contrast. However, electrical measurements had a higher SNR than optical measurements from 840 nm (Figure S7) due to poor optical collection efficiency. Disregarding contrast and laser noise, the measured optical SNR was limited by photon collection efficiency, photons from other defects, and APD detector efficiency and noise. The electrical SNR was limited by noise from a laser-induced background. Photocurrent measurements were not yet limited by TIA noise or noise in the linear power supply bias voltage because tests with a solid-state battery (±<1 μV) showed no reduction in photocurrent variance. Therefore, electrical spin contrast measurements were limited only by electron shot noise from the SiC charge extraction and laser power fluctuations.

Regarding signal improvement (increasing the SNR), electrical readout studies with different bias voltages have been reported^27^ and demonstrate a small increase in the SNR for higher voltages. However, our goal is to perform electrical readout using CMOS-compatible voltages (<5 V). We thus look toward reducing electrode sizes and spacing to reduce the background photocurrent and required voltage, ensuring that the electrode geometries match the illuminated defect region. This reduces the leakage current from nonilluminated regions. Another prospect for contrast improvements toward single-defect PDMR involves reducing the background with partial electrode insulation, as demonstrated in ref 21.

Hahn echo decay was measured and fit with an electron spin-echo envelope modulation (ESEEM) function, showing optical spin coherence times of ∼7 ± 0.5 μs for both the thin film and bulk SiC, which demonstrates a similar *T*_2_ coherence time of the defects in both the bulk and fabricated thin-film SiC on insulator after grinding, polishing, and ICP etching. A fast Fourier transform of the raw Hahn echo decay data reveals frequency components around 150–200 kHz for thin-film and bulk samples (Figure S7). The magnetic field strength of the permanent magnet near the carbide is *B*_0_ ≈ 5 mT, calculated from the Zeeman energy-level splitting in the ODMR data. Electrical measurements of the Hahn echo decay required 10 μs between laser pulses to keep the number of pulses fixed in the modulated RF amplitude envelope while RF pulse spacings were swept. This made measurements greatly susceptible to laser fluctuations. Measurement attempts were made; however, due to low SNR from laser power instabilities, electrical Hahn echo decay results were inconclusive.

Previously, laser wavelengths of 785 ^27^ and 905 nm^21,28^ have demonstrated electrical readout of the V_Si_^–^ spins in SiC. In this work, the excitation wavelength dependence of the Rabi contrast was investigated from 780 to 990 nm electrically and from 780 to 890 nm optically, with results provided in Figure 4B. Optical contrast shows a slight increase from 850 nm, consistent with observations from other optical-contrast wavelength studies of V_Si_^–^ in SiC.^41^ Electrical contrast increases from 780 nm to about 900 nm, likely due to a reduction in non-V_Si_^–^ electrons contributing to the laser-induced background (Figure 4A).

**Figure 4:**
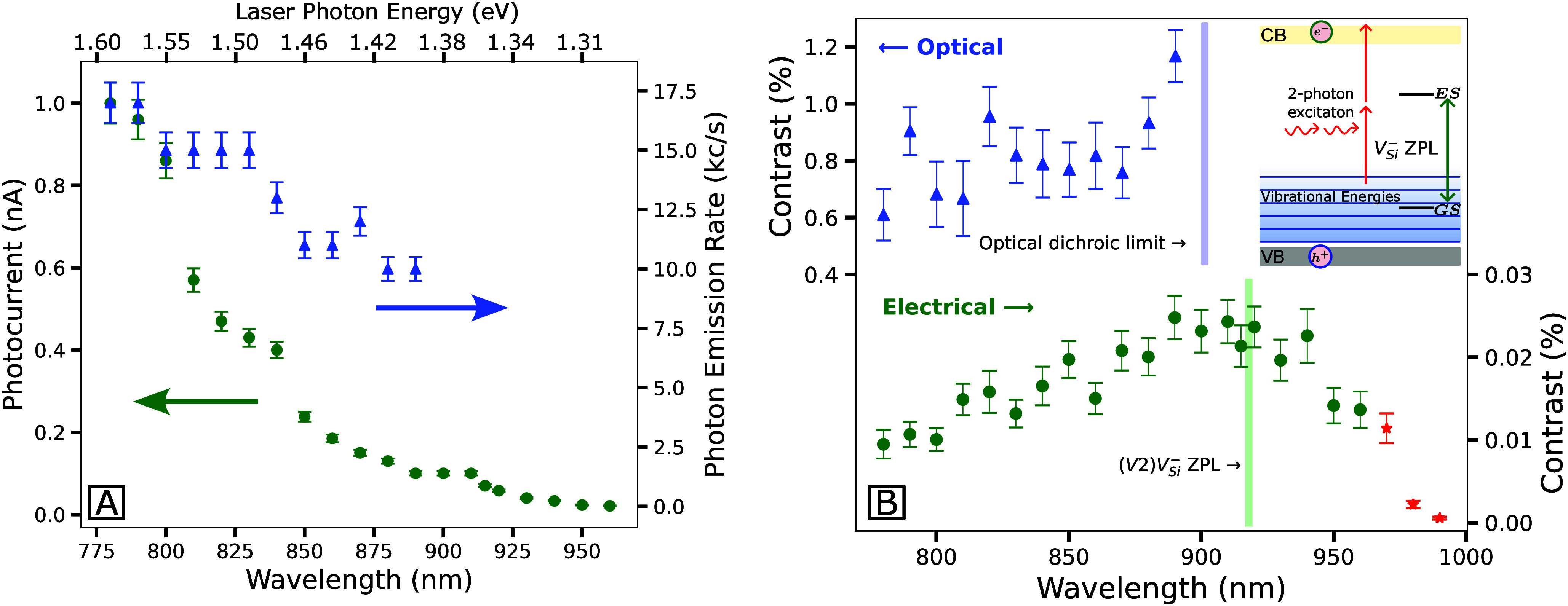
Wavelength dependence of the photocurrent and photon counts (A) and Rabi contrast (B) in 1.3 μm thin-film 4H-SIC on SiO_2_. Each data point in part B is one Rabi period, fitted to a decaying sine function. Rabi is measured electrically with 40 ± 5 mW to 960 nm (green circles) and 40 to 70 mW up to 990 nm (red stars), with the respective photocurrents shown in part A. Photon counts correspond to the optical measurements (blue triangles) performed at 2 ± 0.5 mW. The optical error is directly from the fit, and the electrical error is the 5% variation from experimental stability combined with the fit error. The top-right inset in part B schematically demonstrates the two-photon ionization (red arrows) and phonon-assisted excitation (antistokes), enabling spin contrast beyond the ZPL optical transition.

It is reported that, in some HPSI types (depending on the supplier), V_Si_^–^, carbon vacancy (V_C_), and C antisite–vacancy pair (C_Si_V_C_) are dominant.^42^ The energies required for ionization and excitation of V_C_ are 0.66 and 0.8 eV, respectively,^42^ enabling a contribution to the background photocurrent for this measured wavelength range. C_Si_V_C_ requires energies ranging from 0.72 to 1.07 eV for ionization.^43^ C_Si_V_C_ exhibits calculated ZPLs at 1.16 and 1.33 eV with a strong absorption line at 1.56 eV.^44^ This hypothetically explains the reduction in the defect-contributed background from 780 nm onward (Figure 4A). The wavelength-dependent background photocurrent depends on the defects present in SiC. To reduce the contribution of other defects to the unwanted background photocurrent, it is important to consider the type of SiC being used, what defects may be intrinsically present, and how annealing techniques can remove or create them. Thus, the contrast of the spin system of interest may be improved.

The spin contrast exhibits a positional dependence, likely arising from spatial inhomogeneities in the defect distribution within SiCOI, where the varying distribution of V_Si_^–^ and non-V_Si_^–^ defects leads to regions with differing electron extraction efficiencies. A maximum-photocurrent optimizer was employed to stabilize the experiment against positional drift and produce experimentally repeatable results; however, maximizing the photocurrent does not necessarily correspond to the region of highest contrast. Considering wavelength-dependent charge extraction, shorter wavelengths are more likely to reach one-photon ionization thresholds of certain defects, enhancing their contribution to the photocurrent, while longer wavelengths produce larger spot sizes (e.g., a 31% increase in area at 990 nm compared to 780 nm). From Figure S1, the photocurrent response to a 31% change in the spot-size area within the stable measurement region is 37%. Consequently, the optimizer may converge on different spatial regions for different wavelengths, meaning that the observed wavelength-dependent trends can include variations in the probed sample volume corresponding to the maximum photocurrent. Nevertheless, these effects may be minimal when also considering that the Rabi rate fluctuated by 6% across all wavelengths and the photocurrent reduction is much greater across the wavelengths (1–0.08 nA shown in Figure 4A).

Excitation and photon collection near the ZPL is challenging due to the requirement for spectral filtering. On the other hand, electrical detection does not require collection optics. Thus, a clear spectrum of the electrical Rabi contrast was observed up to 980 nm, with a small signal at 990 nm. This demonstrates for the first time electrical readout of the spin state well beyond the ZPL of V_Si_^–^. We hypothesize that at room temperature this process is enabled via phonon-assisted excitation of the electron from the ground state to the excited state of V_Si_^–^.^45,46^ Previous studies have demonstrated phonon energies persisting from 1.35 to 1.2 eV for V2 V_Si_^–^,^47^ consistent with the demonstrated spin contrast beyond the ZPL. Furthermore, spectral dependence and observation of the electrical Rabi contrast is demonstrated beyond the ZPL due to the threshold energy for ionization and recombination being lower than the defect excitation energy^39^ combined with a band of antistokes vibrational energy contributions,^46^ potentially further assisting the ionization process of V_Si_^–^ (Figure 4B, inset). The significant contrast reduction from 940 nm onward is likely due to a reduction in the excitation of V_Si_^–^ and ionization efficiency, possibly explained by previous works measuring the two-color excited photocurrent of V_Si_^–^,^39^ demonstrating a threshold of 948 nm for the V_Si_^–^-to-V_Si_^2–^ conversion.

Electrical detection provides a wavelength-tolerant readout of the V_Si_^–^ spin state, not limited by wavelength-dependent photon collection optics and devices. Spin-dependent signals were observed electrically for all tested wavelengths. Further investigations to wavelength-dependent spin-state processes can be undertaken using electrical readout.

In summary, we have demonstrated electrical spin readout and coherent control of silicon vacancies in 4H-SiCOI over a wide range of wavelengths, establishing a route toward scalable, CMOS-compatible quantum devices in SiC. By implementing PDMR in a 1.3 μm thin-film SiCOI architecture and comparing bulk SiC samples from the same wafer, we measure similar *T*_2_ coherence times despite undergoing bonding, polishing, and ICP etching, observing clear electrical Rabi oscillations and optical Hahn-echo coherence of ∼7 ± 0.5 μs in both the thin-film and unprocessed bulk material.

Furthermore, we show for the first time that PDMR contrast persists across a wide excitation range (780–990 nm). Excitation wavelengths from 780 nm onward are hypothesized to suppress a background photocurrent from unintended charge sources, improving the contrast and SNR. Wavelengths between 840 and 890 nm demonstrated a Rabi SNR higher than optical measurements due to poor collection efficiency (Figure S6). The contrast extends well beyond the 917 nm V2 V_Si_^–^ ZPL and having optimal contrast around the ZPL ± 20 nm, a regime difficult to access with conventional optical setups. Clear Rabi contrast persists up to 980 nm, with evidence of contrast at 990 nm. The measurement limitation from wavelengths of 970 nm onward is that the photocurrent signal approaches the dark current. Investigating PDMR at cryogenic temperatures could provide insight into using the electrical readout of the spin for measurement of phonon vibronic states.^45,47^

This work combines SiCOI, a platform suitable for large-scale integrated photonic fabrication, with a scalable electrical spin-state detection technique that does not require collection optics. Integrated photonics enables independent spin initialization across a SiCOI wafer, obtainable with optical waveguides. However, on-chip optical readout of these spin states requires fabricated single-photon detectors and demands optical frequency filtering of the initialization laser with a high extinction ratio. Electrical readout, on the other hand, offers a more attractive path for scaling up thousands of on-chip spin detection systems based on patterned metal electrodes. A thin-film platform is essential for guiding light in integrated photonic waveguides. Our results thus provide the first evidence that electrical readout of spin systems (PDMR) is preserved after aggressive thin-film fabrication, a step toward realizing future compact and scalable spin-state detection via electrical readout and waveguides. By combining an array of electrical spin readout electrodes with an integrated photonic network, one could realize a quantum processing chip, useful for distributed quantum computing, enabled by a network of quantum processing modules.^48^ Furthermore, in applications where optical entanglement is not required, an array of spatially separated PDMR electrodes on SiCOI, as shown in this work, can function as individual quantum sensor regions. This pixel array functions as a quantum charge-coupled device, hypothetically yielding a versatile quantum imaging sensor that delivers spatially resolved measurements of electromagnetic fields and temperature, making it applicable for understanding the structure of organic compounds and imaging of electronic devices with submicrometer spatial resolution, extending beyond the limitations of wide-field optical microscopy setups.^49^

## Supplementary Material



## Notes

Spot-size calculation and experiment stabilization, photocurrent characterization, SNR comparison between optical and electrical readouts, Hahn echo fast Fourier transform, SiC-on-insulator fabrication, and experimental hardware and setup (PDF)
