# Supplementary material for: Wavelength-Dependent Electrical Readout of Spin Ensembles in a Thin-Film SiC-on-Insulator Platform
Source: Nano Lett. 2026 Apr 20;26(17):5628–35. doi: 10.1021/acs.nanolett.5c05971 (PMC13154344; doi:10.1021/acs.nanolett.5c05971)
Supplement: Supplementary file 1 [file nl5c05971_si_001.pdf]

Supporting information for 'Wavelength dependent electrical readout of spin ensembles in thin-film silicon carbide on insulator platform"

Alexander Zappacosta*[1], Ben Haylock[1], Paul Fisher[1], Naoya Morioka[2, 3], and Robert Cernansky*[1]

[1]Institute for Quantum Optics, Ulm University
[2]Institute for Chemical Research, Kyoto University, Uji, Japan
[3]Center for Spintronics Research Network, ICR, Kyoto University, Uji, Japan

*Email: alexander.zappacosta@uni-ulm.de, robert.cernansky@uni-ulm.de

April 2, 2026

Spot size and stabilisation

We noticed that the photocurrent and contrast changes due to different spot sizes and positions in the sample; therefore, it was critical to find an optimal point for repeatable results. This specifically matters for the wavelength measurements, so they are not affected by changes in position or spot size.

An objective Airy disk has a spot size measured by the radius from the centre to the first zero of the airy pattern intensity function given by

$$\omega_0 = \frac{1.22\lambda}{2\text{NA}}$$

where λ is the laser wavelength and NA is the numerical aperture of the objective. The spot size is a function of distance z along the illumination direction given by

$$\omega_z = \omega_0\sqrt{1+\frac{z}{z_R}}$$

where

$$z_R = \frac{\pi\omega_0^2}{\lambda}$$

is the the Rayleigh length of the beam. So therefore the beam-radius as a function of z is

$$\omega_z = \frac{1.22\lambda}{2\text{NA}}\sqrt{1+\frac{4\text{NA}^2 z}{1.4884\pi\lambda}}$$

The smallest spot size is when the objective is in focus i.e, $z = 0$. As the sample moves closer to the objective, the surface spot size is estimated by using the distance travelled by the piezo stage from focus. Measuring an electrical confocal scan for a 20 μm x 20 μm area and taking the maximum photocurrent (PC), the change in photocurrent per spot size is shown in Figure S1. We can see there is a peak

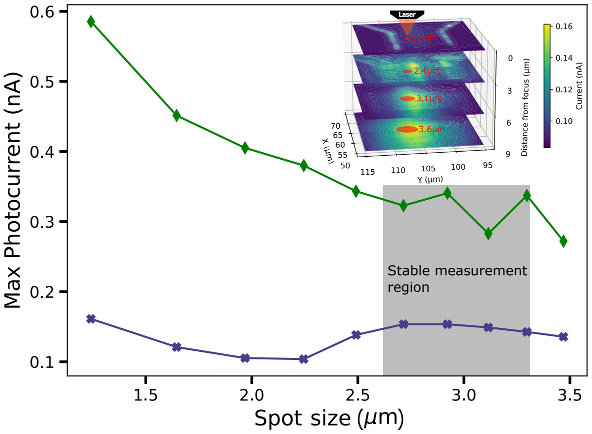

Figure S1: *The maximum photocurrent taken from ten 20 μm × 20 μm confocal scans. The green diamonds are from the 500 μm bulk carbide and purple crosses from the 1.3 μm SiCOI sample each with $\Delta z = 1$ μm and plotted against estimated spot size. Four SiCOI electrical scans are shown in the inset with the spot size as reference and the grey shaded region indicates the spot size for a stable max photocurrent optimiser during measurements.*

photocurrent when the beam is in focus along the electrode edges, and another maximum in between the electrodes when the spot size is 2 to 3 μm.

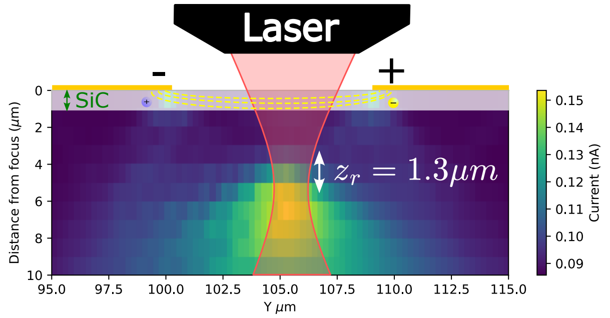

Figure S2: *Electrical confocal scan along the beam propagation z-axis between electrodes in the 1.3 μm thin film on SiO_2 on silicon, measured at 890 nm, 8 V bias and 40 mW. Charge collection is from the thin SiC layer on top. The Rayleigh length is annotated for reference, roughly scaled to the scan region dimensions. Shown is the focal configuration for the data in main text, with an optimiser scanning the sample for maximum photocurrent.*

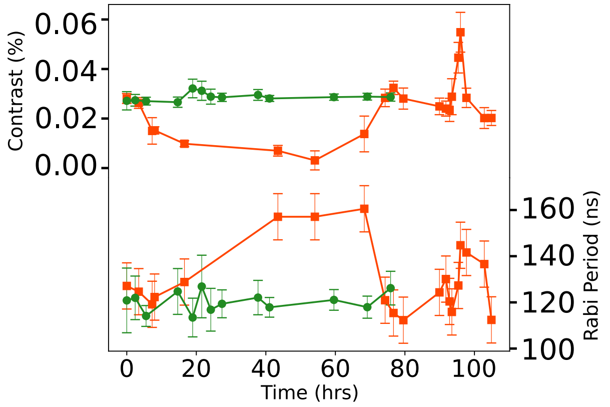

Figure S3: *Contrast and Rabi period was monitored over 3 days with maximum PC optimiser (green circles) and 4 days with no optimiser (orange-red squares). Stabilised contrast standard deviation is ≈ 5% and combined with the fit error of a decaying sinusoidal function used in the main text wavelength measurement error bars.*

Performing a Z-Y scan and measuring the photocurrent, the photocurrent map along the z-direction is shown in Figure S2. The calculated Rayleigh length for 890 nm is $z_r = 1.3$ μm and shown for reference. The top 1.3 μm is the silicon carbide (SiC) and source of charges. This configuration of focusing into the sample was the measurement condition for the data in the main text.

With a larger spot size an optimiser can find the maximum photocurrent within a 7 μm × 7 μm × 0.5 μm translation range every 5 minutes. To test stability, the contrast and Rabi rate was monitored over a period of days to ensure experimental repeatability in the contrast measurements. Figure S3 shows the Rabi contrast and period over 3 days with maximum photocurrent optimiser and 4 days with piezo stage turned off (no optimiser). The maximum photocurrent optimisation algorithm at the stable point shown in Figure S1 has a 5% deviation in measured rabi contrast. With no optimiser the experiment naturally drifts due to temperature fluctuations, finding places with higher and lower contrast. Positional contrast dependence is likely due to having regions of higher V_{Si}^- electron extraction since the distribution of defects is not completely uniform within the silicon carbide on insulator (SiCOI), and some regions could have more or less efficient charge extraction from the V_{Si}^-. One caveat of using the maximum current optimiser is that it does not find the region with maximum contrast; stabilisation to maximum contrast (not photocurrent) requires image processing algorithms to lock to a specific location within a confocal scan image and was not implemented in this work.

Furthermore, by scanning the spot size at a fixed X-Y position, it was also discovered that the relative standard deviation in the photocurrent also changes. By taking 50 photocurrent measurements every 100 ms (five seconds total) for different spot sizes, the variance would decrease and increase along with the photocurrent average from the same 50 data points shown in Figure S4. The variance was monitored before measurements, providing an indication of the measurement noise.

Photocurrent Characterisation

Photocurrent non-linearity was tested for a laser power range of 0 to 40 mW at 890 nm pump wavelength using an 8 V bias across the electrodes, demonstrating a small second-order quadratic relationship shown in Figure S5. The linear component of the fit is plotted as a reference in red. The quadratic relation is a product of the two-photon ionisation process mentioned in the main text; however, this trend varies with position, voltage, and wavelength, most frequently showing a linear trend as most of the photocurrent signal is from non-V_{Si}^- electrons. The percent variance is also seen to increase with laser power, and plateaus from 20 mW with fluctuations possibly due to insufficient settling time of charge extraction from manually sweeping the laser power.

For quantum technology applications, it is also important to check the SNR per wavelength for signal visibility and measurement time. By taking the ratio

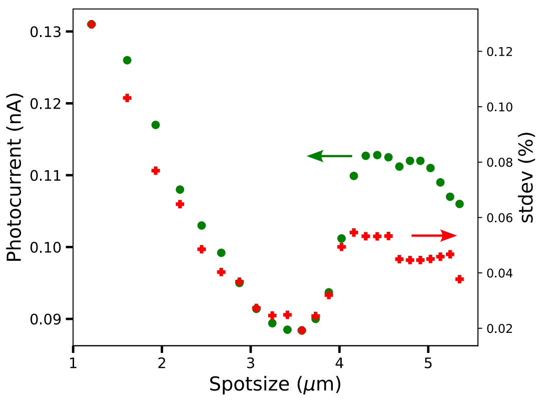

Figure S4: *The standard deviation (red plus) and average (green circle) of fifty photocurrent measurements over 5 seconds plotted for each spot size at 890 nm, 37 mW and 8 V bias. Data taken from one fixed x-y position and beam propagation z-axis translated using a nano-positioning piezo stage. The relative standard deviation is shown to depend on the spot size.*

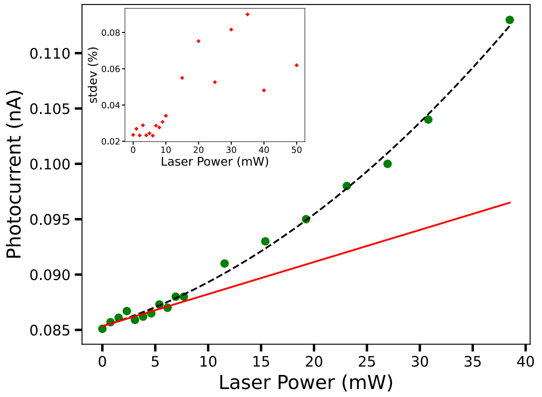

Figure S5: *Photocurrent from the thin film using 890 nm and 8 V bias. The data is fitted to second order polynomial (dashed black line) and the linear component from the fit is plotted as a red solid line. The relative standard deviation shown in the inset is taken from 50 photocurrent measurements over five seconds.*

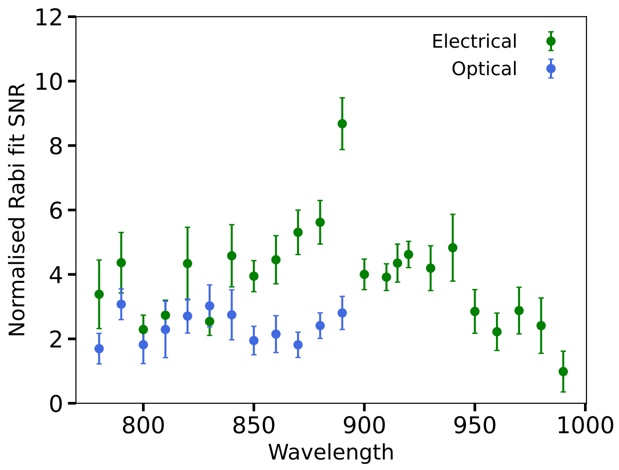

Figure S6: *The signal-to-noise ratio (SNR) per wavelength, extracted from the Rabi oscillation fits in the baseline region, is defined as the ratio of the fitted Rabi contrast to the standard deviation of the residuals (data minus fit) in the wavelength scan shown in the main text. The optical collection efficiency was not optimal due to experimental limitations, which results in a lower SNR; nevertheless, these are the experimental conditions used throughout the main paper.*

of the fitted contrast from the least-squares fit of an exponentially decaying sinusoid C_{fit}, and standard deviation of the residuals from the Rabi fit σ (data minus fit), i.e.

$$\mathrm{SNR_{Rabi}} = C_{\mathrm{fit}}/\sigma$$

and then normalising with measurement time following the method outlined in [1],

$$\mathrm{SNR_{norm}} = \mathrm{SNR_{Rabi}} \times \sqrt{3600/t}$$

where t is measurement time in seconds), the Rabi SNR per wavelength is shown in Figure S6. This method shows an increasing SNR up to 890 nm and consistent reduction from 940 nm for electrical measurements. Although this method of calculating the SNR is not using noise density analysis from the PDMR signal nor direct ratio of the V_{Si}^- electrons and non-V_{Si}^- electrons, it still indicates some wavelength dependent mechanism affecting the spin contrast noise. Further investigation could hypothetically provide insight into using electrical readout of the spin state to observe vibronic states within SiC [2, 3] and hypothetically wavelength-selective vibronic-assisted contrast enhancement or ionisation efficiencies.

SiCOI and electrode fabrication

The SiC wafer is diced into 10 mm x 10 mm chips. A single side polished prime Si + 2000 nm dry/wet/dry SiO2 wafer, 4 inch, 525 µm thick, from Microchemicals GmbH is cleaved into 15 mm × 15 mm chips. Both the SiC and Si/SiO2 chips undergo a thorough cleaning procedure, with Piranha, RCA1, and RCA2 cleaning, followed by an oxygen plasma surface preparation. The chips are placed in a wafer bonder (SüSS SB6e), Under vacuum in the wafer bonder the chips are heated to 450° C and pressed together with a downward force of 1500 N for two

hours. The chips are then cooled, the downward force released, and returned to atmospheric pressure. This bonded stack then undergoes grinding and polishing to thin the SiC to approximately 40 µm. This consists of a grinding step with a D80 diamond grinding wheel (Dopa Diamond Tools), followed by three polishing steps with successively finer diamond slurry (6 µm, 1.5 µm, 0.25 µm). Finally, the film undergoes dry etching with ICP-RIE to thin the film from 40 µm down to the final desired thickness of 1.3 µm.

The sample was cleaned in piranha solution and electrode designs were patterned with a UV laser-lithography system and AZ10XT photoresist. After development, the sample underwent an oxygen plasma clean to remove surface contaminants. Chrome was deposited as an adhesion layer using electron beam evaporation with 10 nm thickness and 150 nm of Gold for conduction. Liftoff was possible after soaking for 2 hours in 80° C NEP then sonicated for 4 seconds. Allresist SX AR-N 8400, hydrogen silsesquioxane (HSQ) was spun to 1.4µm thickness, patterned leaving windows for bonding pads, and baked at 250° C for 30 mins to cure on top of the SiCOI surface. The sample was then glued and wire bonded with 25 µm aluminium wire to a 50 Ω impedance matched PCB. A 50 µm enamelled copper wire was strung perpendicular to the electrode tips and soldered to the same PCB.

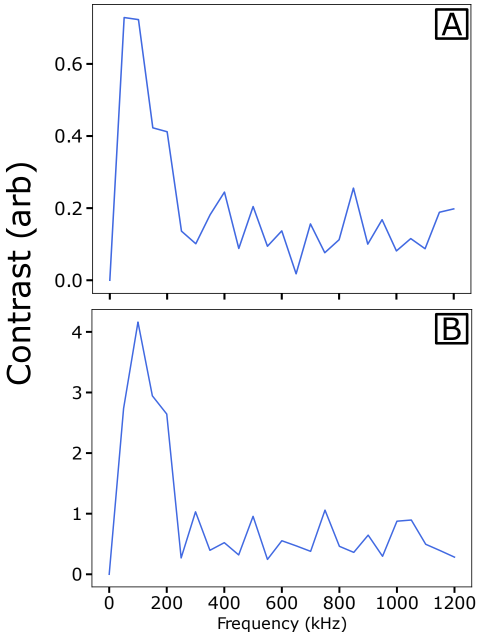

Figure S7: *Fast Fourier Transform of the raw T2 Hahn echo decay for the thin-film (A) and bulk (B), demonstrating broad frequency components around 150kHz to 200kHz. The magnetic field strength at the defects is ≈ 5mT.*

Hahn Echo decay Fast Fourier Transform

The Fast Fourier Transform of the raw data from the T2 hahn echo decay is shown in S7. The origin of such coupled frequencies to the electrons is unknown; however, it is hypothesised to originate from impurities within the bulk of the material, given the prominence of the signal. Provided the shape is not a consistent Lorentzian, multiple frequency components could be present.

Experimental setup

The sample was mounted to a 3-axis piezo stage (Piezoconcept LT3-200) to move the sample into the focal positions of the microscope. Measurements were performed using a fixed path home-built confocal microscope equipped with a 0.9 NA air objective (Zeiss EC Epiplan-NEOFLUAR 100x/0.9 DIC) for laser focusing and fluorescence collection. Defect excitation was achieved using a fully automated Sirah Ti:Sa CW ring laser, modulated using an acousto-optic modulator (CASTECH CAOM-200-015-TEC-780-950-AF-A17). The collection path was filtered with two 900 nm longpass filters and one 1000 nm shortpass filter (Thorlabs FELH0900, FESH1000). Filtering the laser and collection paths using a 900 nm dichroic mirror (Thorlabs DMLP900). Fluorescence was focused into a fiber coupled APD (Excelitas SPCM-AQRH-14-FC-ND) connected to a National Instruments DAQ (NI USB-6363) for data collection.

One gold electrode was connected to the signal terminal of a DLPCA-200 transimpendence amplifier (TIA) from Femto, set to 10 Hz low pass filter in DC coupling mode for an absolute current measurement. Connections were made using a standard coaxial cable for signal shielding and BNC to screw terminal to split the signal and ground terminals of the TIA.

The second gold electrode was connected to either the positive or negative output terminal of a Siglent SPD3303 linear power supply for voltage biasing. Polarity was chosen by sweeping voltage ranges of 0 to 9 V while trying to maximise laser-induced photocurrent signal to dark signal ratio. The opposite power supply terminal was connected to an un-anodised aluminium breadboard, which served as a fully isolated ground plane sitting on a rectangle of rubber, on which the whole confocal system stood, this acted as the completely isolated ground plane. The ground plane was then directly connected to the building ground. This ground was shared with all measurement devices in the experiment, reducing the possi-

bility of ground loops.

The TIA ground terminal was directly connected to the breadboard ground plane, the metal casing of the uninsulated TIA sat on the breadboard shared also with the PCB and RF ground. EMF shielding was achieved by building a cardboard box wrapped in aluminium foil Faraday cage grounded to the same plane.

RF pulses were delivered using a vector signal generator (Siglent SSG3021X-IQE), amplitude modulated with a fast switch (ZASW-2-50DRA+) and amplified

(Mini-Circuits, ZHL-5W-1+). The AOM and RF pulses were synchronised using a Swabian Instruments Pulse Streamer. Rabi and pulsed PDMR sequences were streamed continuously for each randomised PDMR frequency (PDMR) or duration (Rabi), keeping the number of laser pulses the same integer number for all RF durations within an 8 Hz square wave period. This 8 Hz envelope was used for digital RF amplitude modulation signal and reference points. Analog voltage measurements were collected from the TIA directly using the DAQ, triggered at the sampling rate limit of the analog to digital converter (500 ns). Data from the first and second half of the 8 Hz envelope was programmatically separated into signal and reference (see Figure S8 top), separately integrated over the window of highest amplifier SNR (see Figure S8 bottom) to get a single value for signal and reference then divided for contrast .

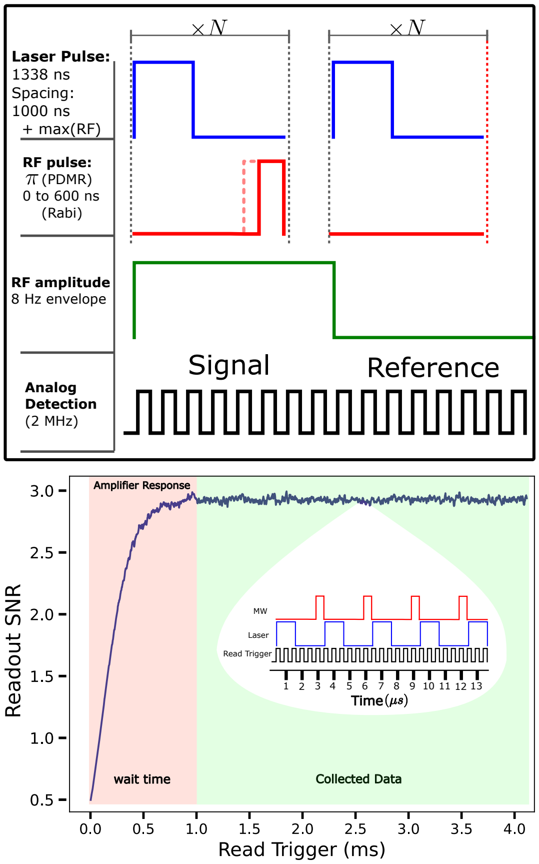

Figure S8: *Schematic of the PDMR pulse sequence (top) with RF amplitude modulation envelope. Pulse sequence was repeated N times, where N = (0.5*envelopePeriod) / (laserDuration + RFSpacing + max(RFPulseDuration)), ensuring N is an integer. Data was integrated over each half envelope period for signal and reference. Bottom showing the wait time of the amplifier to account for the 1kHz response time then integrated over the repeating sequence while modulating the RF amplitude.*

References

(1) Niethammer, M.; Widmann, M.; Rendler, T.; Morioka, N.; Chen, Y.; Stöhr, R.; Hassan, J. U.; Onoda, S.; Ohshima, T.; Lee, S.; Mukherjee, A.; Isoya, J.; Son, N. T.; Wrachtrup, J. Coherent electrical readout of defect spins in silicon carbide by photo-ionization at ambient conditions. *Nature Communications* **2019**, *10*, 5569.

(2) Udvarhelyi, P.; Thiering, G.; Morioka, N.; Babin, C.; Kaiser, F.; Lukin, D.; Ohshima, T.; Ul-Hassan, J.; Son, N. T.; Vučković, J.; Wrachtrup, J.; Gali, A. Vibronic States and Their Effect on the Temperature and Strain Dependence of Silicon-Vacancy Qubits in 4H-SiC. *Physical Review Applied* **2020**, *13*, 054017.

(3) Shang, Z.; Hashemi, A.; Berencén, Y.; Komsa, H.-P.; Erhart, P.; Zhou, S.; Helm, M.; Krasheninnikov, A. V.; Astakhov, G. V. Local vibrational modes of Si vacancy spin qubits in SiC. *Physical Review B* **2020**, *101*, 144109.
